# Kounis Syndrome together with Myocardial Bridging Leading to Acute Myocardial Infarction at Young Age

**DOI:** 10.1155/2011/490310

**Published:** 2011-10-11

**Authors:** Ilker Murat Caglar, Alper Vural, Fatma Nihan Turhan Caglar, Murat Ugurlucan, Osman Karakaya

**Affiliations:** ^1^Department of Cardiology, Bakirkoy Education and Research Hospital, Istanbul, Turkey; ^2^Institute of Cardiology, Istanbul University, Istanbul, Turkey; ^3^Department of Cardiovascular Surgery, Duzce Ataturk State Hospital, Duzce, Turkey

## Abstract

Kounis syndrome, also named as “allergic angina syndrome,” is a diagnosis in which exposure to an allergen causes mostly coronary spasm and rarely plaque rupture, resulting in ischemic myocardial events. Myocardial bridging is defined as an intramural segment of a coronary artery and its systolic compression by overlying fibers. Myocardial bridging generally has a benign prognosis and mostly affects the mid portion of left anterior descending coronary artery. However, some cases with myocardial ischemia, infarction, and sudden death have also been reported. A 17-year-old boy presented to the clinic with acute anterolateral myocardial infarction after having first dose of clindamycin and diagnosed as Kounis syndrome. Further diagnostic workup of the patient showed myocardial bridging at the mid left anterior descending artery. In this report, we present the combination of Kounis syndrome and myocardial bridging leading to myocardial infarction at young age.

## 1. Introduction

Myocardial bridging (MB) is called when a segment of a major coronary artery courses intramurally. The intramural part is named as the “tunnelled segment,” and mostly the mid portion of the left anterior descending coronary artery (LAD) is affected [1, 2]. There is a temporary systolic narrowing of the coronary lumen, which sometimes causes symptoms usually in middle-aged man, as typical or atypical chest pain related or unrelated with excercise [3–5]. It is usually a benign anatomical variation; however, in some cases myocardial ischemia, infarction, arrhythmias, and sudden cardiac death may also occur [6, 7]. 

Myocardial ischemia signs and symptoms accompanied by allergic or hypersensitivity and anaphylactic or anaphylactoid reactions are defined as Kounis syndrome (KS) [8]. It is also referred as a new reason for coronary artery spasm [9, 10]. There are very rare well-defined Kounis cases in the literature. Many drugs, environmental factors, and conditions that can cause allergic reactions may also be the causes for KS [9, 10]. So far two types of KS have been defined [10]. Type 1 is seen in patients who have normal coronary arteries, without predisposing factors for coronary artery disease. Mast cell activation and vasospasm are the main causes, and this type represents manifestations of endothelial dysfunction and microvascular angina. Type 2 variant is seen in patients who have dormant atheromatous plaques. Mediators of acute allergic episode enhance plaque erosion which may progress into myocardial infarction (MI) [10, 11]. Prognosis of patients with Type 1 KS is better than Type 2 cases. Prognosis depends on the magnitude of the allergic reaction, patient's sensitivity, comorbidities, allergen concentration, and allergen's portal of entrance to body [11]. The most frequent symptoms are retrosternal chest pain, dyspnea, palpitation, weakness, nausea, urticaria, itchiness, sweating, and hypotonia [11]. 

In this report, we have a case Type 1 KS together with myocardial bridging at the mid portion of the LAD leading to acute anterolateral myocardial infarction in a 17-year-old male patient.

## 2. Case Report

A 17-year-old boy presented to the emergency unit complaining of retrosternal chest pain radiating to his left shoulder and back for one hour. Four days ago, he has started to receive penicillin treatment due to acute tonsillitis, and, on the fourth dose of penicillin, he had an episode of severe itching, nausea, and vomiting. It was accepted as penicillin allergy, and the treatment was changed to clindamycin therapy. One hour after the first dose of clindamycin, the retrosternal chest pain has started. 

**Figure 1:**
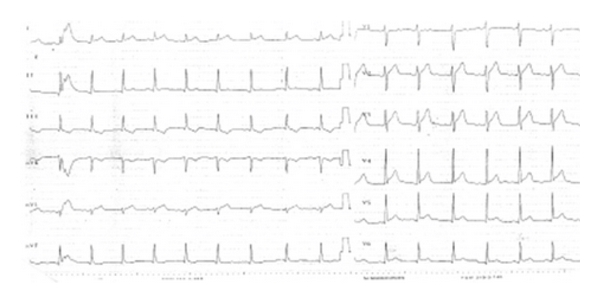
Anteroseptal ST elevations on leads DI, aVL, V2 to V6, and reciprocal ST depressions on leads DIII, aVF indicating acute anterolateral myocardial infarction.

His electrocardiogram (ECG) on admission indicated acute anterolateral MI with ST segment elevations on leads DI, Avl, and V2 to V6 and also reciprocal ST depressions on leads DIII and aVF (Figure 1). He had no risk factors for coronary artery disease, and his physical examination was unremarkable, but his blood tests showed elevated troponin levels as 1.4 ng/mL (normal range 0.00–0.11 ng/mL) and leucocytosis and eosinophilia. His echocardiography showed no pathologic signs of pericarditis, but he had slightly hypokinetic mid and apical anterior segments of left ventricle.

**Figure 2:**
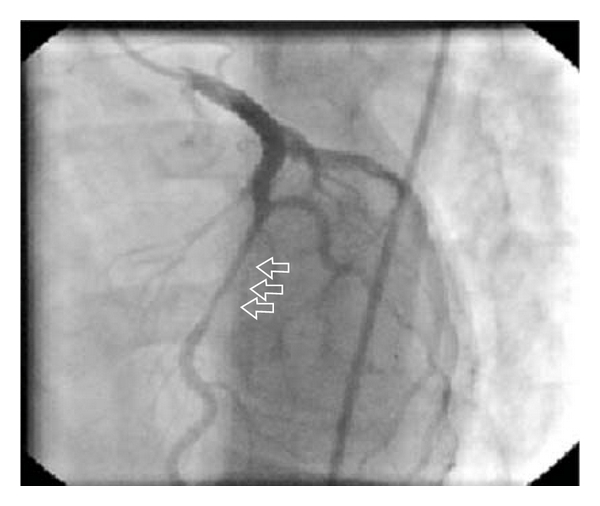
Myocardial bridging at the mid portion of the LAD and narrowing during systole.

**Figure 3:**
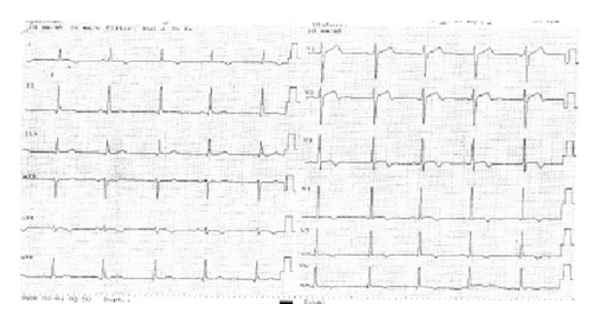
Normal resolution phase of anterolateral myocardial infarction as biphasic T waves on leads V2 to V6 and DI, aVL.

He was taken to the invasive laboratory, and coronary angiography was performed. His angiogram resulted as totally normal coronary arteries except at about 15 mm in length myocardial bridging at the mid portion of the LAD (Figure 2). No further interventions were performed, and he was taken to intensive care unit. Combination of beta-blocker, acetyl salicylic acid and prednisolone therapy was started. His symptoms dissolved in half an hour, and during followup no symptoms repeated. His troponin levels elevated up to 18 ng/mL as peak and started to decrease then after gradually. His ECG findings resolved in the following days as the ST elevations and reciprocal ST depressions returned to baseline and the biphasic T waves observed as expected (Figure 3). He was discharged on the fifth day, and, at the 3- and 6- month visits, he was free of cardiac complaints.

## 3. Discussion

In 1991, regarding Kounis syndrome, the first case was reported by Kounis and Zarvas as “allergic angina syndrome.” Constantinides declared that even nonserious allergic reactions could provoke atherosclerotic plaque rupture, in 1995. In 1998, Braunwald pointed out that histamine and leukotriene-like mediators released during allergic reactions contracted the coronary blood vessels [11]. Myocardial infarction due to amoxicillin as an allergen was first described in 1950. To our knowledge, there is no report on clindamycin causing myocardial ischemic syndromes in the literature. 

Mast cells, the key elements of allergic reactions, are located between cardiomyocytes and around coronary arteries in healthy people. Larger amounts are present at atherosclerotic vessels. They play major role in allergic and anaphylactoid reactions. After allergen exposure, antigen-anticor complexes or the allergen itself directly activates mast cells [9, 11]. Mediators like histamine, leukotrienes, neutral proteases, and platelet-activating factor are released locally and to the systemic circulation by mast cell degranulation to constrict the coronary arteries. 

Myocardial bridging is usually an incidental finding at coronary angiography, but sometimes the patients may be admitted to the emergency units with typical or atypical angina pectoris, ST elevation, or non-ST elevation MI and very rarely with sudden cardiac death in the presence of myocardial bridging [12, 13]. It was first recognised at an autopsy by Reyman in 1737 and first described angiographically by Portmann and Iwing in 1960 [14, 15]. The estimated incidence varies from 1.5% to 16% when assessed by coronary angiography; however, it's much higher in autopsy series as up to 80% [1, 16]. In the vast majority of the cases, angiographic localization of the bridge is on the LAD. The right coronary artery or the circumflex artery involvements are very rare [17–19]. 

Myocardial bridging is usually a clinically silent condition, and the clinical significance of the bridge is determined by the anatomy of the tunnelled segment, concomitant vaso-constrictive situations, and atheromatous changes. During systole, contraction of the overlying myocardium compresses the underlying coronary segment; this compression may persist into the diastole, in which the majority of coronary blood flow occurs normally. Increased heart rate, shortening of diastole, increased myocardial contraction and flow velocity, and all causes leading to coronary spasm like exercise, drugs, or allergic reactions can cause ischemia in patients with myocardial bridging [20, 21]. The myocardial infarction in our case may be related to the additive effect of coronary vasospasm of Type 1 Kounis syndrome and the existing myocardial bridging on the mid LAD. 

In symptomatic patients with myocardial bridging, the management is usually medical rarely surgical or invasive. Available medications include beta-blockers and calcium channel blockers. These drugs have the negative inotropic properties that slows the heart rate and elongates the diastolic phase to obtain adequate time for the coronary arteries to maintain the blood flow. Nitrates should generally be avoided because they can increase the angiographic narrowing due to the increased heart rate and can lead to worsening of the symptoms. Surgical treatment by dissecting the overlying myocardial fibers (myotomy) or coronary artery bypass should be limited to patients with severe symptoms or recurrent ischemia that persist despite optimal medical treatment. Coronary stenting may be another treatment option in retractable cases; however, the restenosis rates and complications during the procedures limit their frequent usage [22]. Our patient was successfully treated conservatively with beta-blocker, aspirin, and immunosuppressive therapy.

## 4. Conclusions

To the best of our knowledge, it is the first case report in the literature that Type 1 Kounis syndrome overlapping with preexisting myocardial bridging and causing acute ST elevation MI in a 17-year-old male patient right after taking a clindamycin tablette. It should be kept in mind that, although the myocardial bridging is considered a safe and simple condition, with the additive vasoconstrictive effects of allergens, drugs, or the patients, it can cause ischemia and infarction.
